# Supplementary material for: Challenges to nutrition management among patients using antiretroviral therapy in primary health ‘centres’ in Addis Ababa, Ethiopia: A phenomenological study
Source: PLoS One. 2021 Jun 17;16(6):e0250919. doi: 10.1371/journal.pone.0250919 (PMC8211200; doi:10.1371/journal.pone.0250919)
Supplement: S1 File — (PDF) [file pone.0250919.s002.pdf]

APPENDIX

Appendix I

Informed Consent Form of Patients living with HIV

TITLE OF STUDY: Challenges to Nutrition Management among People Living with HIV on ARV medication

PRINCIPAL INVESTIGATOR: Helen Ali

ADVISORS :Dr. Kassa Daka, Mr. Befekadu Bekele and Mengistu Meskele

You will be asked to take part in this research study because you are currently living with HIV, and we want to find out more about how your diet or eating has changed since receiving this diagnosis. The purpose of this study is to find out if people living with HIV need to make a lot of changes to their diet, eating patterns, or nutritional intake, and to learn about any problems or issues they face . The research will in three health centers in Addis Ababa, Ethiopia.

You will be asked to spend about 50 minutes participating in an interview. You will be asked a series of questions about a variety of topics, including problems you have faced in managing food or diet since being diagnosed with HIV. The importance of food or food problems in your life right now, and skills related to preparing food or managing diet and nutrition. The Interview will be recorded on audiotape. These tapes will not identify people in particular except for their positions. The tapes will be reviewed only by qualified research staff.

You do not need to talk about anything that makes you uncomfortable. It is not possible to identify all potential risks in research procedures, but the researchers have taken reasonable safeguards to minimize any known and potential.

Your participation will also help in the development of a workshop for clinic staff that will give information to them about what kinds of things to discuss with patients about food, as well as food challenges that patients might encounter and how to deal with them.

You should not feel that there is any pressure to take part in the study, to please the investigator or the research staff. You are free to participate in this research or withdraw at any time.

All information gathered during your participation will be used for research purposes only, and will only be seen by members of the research staff. The Wolaita Sodo University School of Public Health Human Nutrition Department may inspect study records. Which they are groups that monitor research to make sure that they are safe for participants. Therefore, your responses will remain secure and anonymous. We may publish what we learn from this study. If we do, we will not let anyone know your Personal data. We will not publish anything else that would let people know who you are.

Before you decide whether to accept this invitation to take part in the study, please ask any questions that might come to mind now. Later, if you have questions about the study, you can contact the investigator, Helen Ali:. Phone 0909679708. We will give you a copy of this consent form to take with you.

Your signature acknowledges that you have read the information stated and willingly sign this consent form. Your signature also acknowledges that you have received, on the date signed, a copy of this document containing 3 pages.

____ _____________________

Signature of person agreeing to take part in the study

____ _____________________

Name of person providing information to participant Date

Appendix III

Demographic Questionnaire

Thank you for coming today. We are interested in finding out what people know the experiences in which you have been challenging as an HIV patient on ART. We have a question guide which guides you to discuss some areas. We have a few background questions before we start with the interview. Please answer the following questions about yourself:

1. Age? ______
2. Gender M ______ F ______
3. Marital status ________
4. Residence ______
5. Occupation ______
6. When were you diagnosed with HIV and started the drug? _________

Patients Question Guide

Thank you for agreeing to participate in this interview. My name is Helen Ali. I came from Wolaita Sodo University School of Public Health. For those of you who have never done this kind of survey before, I want to tell you that it is a research technique commonly used in social science research to gather data from informed sources. Your answers to our questions will not be considered 'right' or 'wrong'. They are merely information that you will supply based on your experiences, observations, or feelings. As mentioned before in the consent forms, this research is interested in finding out how and why people living with HIV have been challenged in managing their nutrition. I am going to ask you starting from the day you have been diagnosed with the disease. I ultimately want to understand what was your experience in managing g your nutrition kind of information or help you could best use to manage your own nutrition.

Before we begin,

Let you introduce your name, why don't we talk about ourselves anything which you want to talk about. You also tell me how long you have lived in this area, and what your 2 most favorite foods are?

Area of assessment	Question Guide
Starting from First Moment 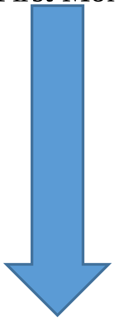	○ When and how you know about your HIV status? Tell me about your feeling at the time?<br>Probing: How was your feeling, emotion negative or positive?
	○ Tell me what it meant to you when you discovered you had HIV?<br>Probing: Regarding changes in your life?<br><br>○ How about your family perception regarding the status you discovered?<br>Probing: Tell me what it meant to you their support,<br>And/or any other support?
Current situation	

- Describe to me how your disease affected your eating pattern change?

  Probing: What are the changes you made?

  What does it mean to you having the eating habit change ?

- Tell me about the challenges you encounter to manage proper eating behavior?

  Probing: What helped you to manage and recover?

  What was your experience while the time of weight change ?

- How do you see the challenge of taking medication in relation with your eating?

  Probing: What helped you to take medication?

  How do you describe the challenges you face to take medications (adherence facilitator and barrier)

- Tell me about your experience with the services regarding nutrition management at the health center ?

  Probing: Education?

  Knowledge?

- Do you want to add anything else?

Wrap-up. 20 minutes. Here’s a summary of the main things we talked about today

Does that sound like everything is discussed? Is there anything else you would like to add?
