# Supplementary material for: Challenges to nutrition management among patients using antiretroviral therapy in primary health ‘centres’ in Addis Ababa, Ethiopia: A phenomenological study
Source: PLoS One. 2021 Jun 17;16(6):e0250919. doi: 10.1371/journal.pone.0250919 (PMC8211200; doi:10.1371/journal.pone.0250919)
Supplement: S2 File — (PDF) [file pone.0250919.s003.pdf]

**<u>የፈቃደኝነትውል</u>**

**የጥናቱርዕስ፡-**የኤች አይ ቪ መድሃኒት በሚከታተሉ የኤች አይ ቪ ኤድስ ህሙማን ሥነ ምግብ ቁጥጥር በሚመለከት

**የጥናቱአጥኚ፡-** ሔለን አሊ የሥነ ምግብ ተማሪ

**አማካሪአጥኚዎች፡-** ዶ/ር ካሳ ዳካ፣ አቶ በፍቃዱ በቀለ እና መንግሥቱ መስቀሌ

በዚህ መጠይቅ ላይ የምትሳተፉት እርሶ ኤች አይ ቪ በደምዎት ውስጥ ስላለ እና መድሃኒቱን እየተከታተሉ ስለሆነ ነው፡፡ ኤች አይ ቪ በደምዎት እንዳለብዎት ከተነገሩበት ጊዜ ጀምሮ በአመጋገብዎት ላይ ያለውን ተለምዶ እና ለውጥ ማየት ፣የዚህ ጥናት አላማ በአመጋገብ ለውጥ ያስፈልግ እንደሆነ፣ ያጋጠሙ ችግሮችን ማየት ይሆናል፡፡ ጥናቱ የሚካሄደው በሶስት ጤና ጣቢያዎች ነው፡፡

እዚህ ጥናት ቃለ መጠይቅ ላይ ለመሳተፍ 35 ደቂቃ ብቻ የሚሰው ይሆናል፡፡ በተለያዩ ርዕሶች ዙሪያ እና ያሳለፉትን ችግሮች ጨምሮ ተከታታይ ጥያቄዎችን ይጠየቃሉ፡፡ የምግብን አስፈላጊነት አሁን ባሉበት ሁኔታ እንዴት አድርገው ምግቦትን እንደ ሚቆጣጠሩም ይጠየቃሉ፡፡ ይህ ቃለ መጠይቅ የድምፅ መቅጃ የሚቀዳ ይሆናል፡፡ እነዚህ መቅረጸ ድምጾች የሰዎችን የስራ ድርሻ ብቻ የሚገልጹ ሲሆን ማንነቶትን የሚገልጽ ሁኔታ አይኖርም፡፡ እነዚህ መቅረጸ ድምቆች የሚታዩት በባለሞያ ለዚሁ ለጥናት አላማ ብቻ ይሆናል፡፡

እርስዎ ፈቃደኛ ያልሆኑበ ትንርዕ ሰጉዳይ ያለ ማውራት ባለሙሉመ ብትነዎት፡፡ ሁሉንም ችግሮች ለይቶ ማየት ቢከብድም የጥናቱ ተሳታፊዎች እርሶ ላይ ለሚሆነው የሚታዎቅ ነገር ሃላፊነት የሚዎስዱ ይሆናል፡፡ ይህ የሚሰጡት መረጃ ለጤና ባለሞያዎቹ የእናንተን የአመጋገብ ችግር እና እንዴት እንዳለፋችሁት በግልጽ የሚያሳይ ይሆናል፡፡

በዚህ ጥናት ላይ ለመሳተፍ ግፊት እንደማይደረግቦትና በማንኛውም ሰዓት የመሳተፍ የመተውም መብት አለዎት፡፡ የሚሰበሰቡት መረጃዎች በአጠቃላይ ለጥናታዊ ምርምር ብቻ የሚወሰዱ ነው የሚሆነው፡፡ ይህንን ጥናት የወላይታ ሶዶ ዩኒቨርሲቲ የጥናት እና ምርምር ዘርፍ የሚያየው ሲሆን ይህ ጥናት እየተካሄደ እያለ የሚቆጣጠሩ አካላትም የሚመለከቱት ይሆናል፡፡ ስለዚህ እርሶ የሚሰጡን መረጃ በሚስጥር ይጠበቃል፡፡ ይህ ጥናታዊ ጽሁፍ የተገኘውን ችግር ቢታተም ምንም አይነት ግላዊ የእርሶ የሆነ መረጃ አይለቀቅም፡፡

እዚህ ጥናታዊ ጽሁፍ ላይ ከመሳተፍዎ በፊት መጠየቅ የሚፈልጉት ጥያቄ ካለ መጠየቅ ይችላሉ ምናልባትም ጥያቄዎቹን በኃላ መጠየቅ ቢፈልጉ ጥናቱ የሚያጠኑ ሔለን አሊ ስልክ ቁጥር ፡- 0909679708 ብለው ይደውሉ።
ፊርማዎት የሚያረጋግጠው ከላይ የተገለጹትን መረጃዎች እንዳነበቡ እንዲሁም ለመሳተፍ ፈቃደኛ መሆንዎትን ይሆናል. የዚህን ፈቃደኝነት ወረቀት ግልባጭ መውሰዶትን አይዘንጉ። እናመሰግናለን!

ጥናቱ ላይ ለመሳተፍ የፈቀደው ፊርማ ና ቀን፡ …………

ጥናታዊ ጽሁፉ ላይ መጠይቅ አሳታፊ ፊርማ ና ቀን … ….

**ግላዊ መጠይቅ**

ስለመጡ እያመሰገንን የኤችአይቪ ታማሚዎች የሚኖራቸውን የአመጋገብ ቁጥጥር በሚመለከት የሚካሄደው መጠይቅ ላይ ለመሳተፍ ፈቃደኛ ስለሆኑ እጅግ በጣም አመሰግናለሁ፡፡ በመጠይቁ መጨረሻ መረዳት የምንፈልገው እንዴት ኤችአይቪን በሥነምግብ መቆጣጠር እንደምንችል ይሆናል፡፡ መጠይቁን ከመጀመራችን በፊት እንደ መግቢያ የተወሰኑ ጥያቄዎች የሚኖሩ ይሆናል፡፡

1. እድሜ
2. ጾታ፡- ወንድሴት
3. የጋብቻሁኔታ
4. አድራሻዎት
5. መቼ ነው ኤች አይ ቪ እንዳለብዎት ያወቁት እና መድሃኒቱን የጀመሩት
6. የሥራ ሁኔታ

መጠይቅ

መግቢያ፡-እዚህ ቃለ መጠይቅ ላይ ለመሳተፍ ፈቃደኛ በመሆንዎት እጅግ በጣም አመሰግናለሁ፡፡ ስሜ ሄለን አሊ ይባላል፡፡የመጣሁት ከወላይታ ሶዶ ዩኒቨርሲቲ የማህበረሰብ ጤና ትምህርት ዘርፍ ነው ከዚህ በፊት እንዲህ አይነት ጥናት ላይ ተሳትፈው የማያውቁ ከሆነ ይሄ ጥናት የጥናታዊ ጽሁፍ አይነት ሲሆን በአብዛሃኛው በማበራዊ ሳይንስ መረጃን ለመሰብሰብ የምንጠቀምበት ነው፡፡ ይህንን ጥያቄዎች ሲመልሱ ትክክል ወይም ስህተት የሚባል ድምዳሜ አይኖረንም፡፡ ሃሳቦቹ የናንተው ያሳለፋችሁት ፣ያያችሁት እንዲሁም ደግሞ ውስጣዊ ስሜታችሁን ብቻ የሚገልጽ እንደሆነ ተደርጎ ይወሰዳል፡፡ እንደተገለጸው ኤች አይ ቪ በደማቸው ያሉ ሰዎች ስለአመጋገባቸው እና ያሳለፉትን ስነ ምነግብን የመቆጣጠር ተግዳሮቶች ማጥናት ሲሆን ከናንተም የሚጠበቀው ያሳለፋችሁትን የህይወት ተግዳሮት ስነ ግበንን ከመከታተል አንጻር ማብራራት ይሆናል ለዚህም ይመራ ዘንድ አጋዥ ጥያቄዎችን እንደሚከተለው እያቀረብኩኝ አብረነብ ቆይታ እናደርጋለን፡፡ መጠይቁ በአማካይ 30 እስከ ደቂቃበላይየሚወስድአይሆንም፡፡በድጋሚ አመሰግናለሁ፡፡

| Area of assessment | Question Guide |
| --- | --- |
| Starting from<br><br>First Moment<br><br>Current situation | 1. መቼና እንዴት ነበር ህመሙ እንዳለቦት ያወቁት፤ እንዴት ነበር የነበሮት ስሜት፤ እንዴት ተቀበሉት<br>አ፤ጥ፡ ስሜትዎት፤ የነበረዎት አቀባበል እንዴት ይገልፁጸታል<br>2. ለእርሶ ምን ማለት ነበር ከህመሙን ሲቀበሉት<br>አ፤ጥ፡ ለውጦች ምን ምን ነበሩ<br>3. የቤተሰቦት ህመሙን ቅበላ እንዴት ነበር;<br>አ፤ጥ፡ እገዛቸው ምን ይመስል ነበር<br>4. ህመሙ የአመጋገብ ለውጥ እንዲያመጡ ምን አስተዋጾ አርጓል<br>ምን ምን ለውጥ አረጉ እንዴት ነበር ሁኔታውን ቢያብራሩልኝ፤ ለውጥ ማድረግ በእርሶ እንዴት ይብራራልኝ<br>5. ስለ አመጋገገብ ተግዳሮቶች ያብራሩልኝ<br>አ፤ጥ፡ ምን ነበር የረዳዎት፡ ምን ማለት ነው የምግ ለውጥ ማድረግ በእርሶ ዘንድ<br>6. መድሃኒት መከታተል ላይ ያለው ተግዳሮት ነእንዴት ያዩታል<br>አ፤ጥ፡ ምን ረዳዎት መድሃኒት እንዲከታተሉ፡ ምን ምን ነበሩ ያዩአቸው ችግሮች<br>7. በጤና ጣቢያ ውስጥ ያለውን የስነ ምግብ ክትት እንዲሁም ሌሎች ከዚሁጋ የተያያዙ አገልግሎቶችን እንዴት ያዩታል<br>አ፤ጥ፡ ትምህርት ፤ እውቀት፤ እርዳታ<br>. መጨመር የምትፈልጉት ነገር አለ |
