# Supplementary material for: Challenges to nutrition management among patients using antiretroviral therapy in primary health ‘centres’ in Addis Ababa, Ethiopia: A phenomenological study
Source: PLoS One. 2021 Jun 17;16(6):e0250919. doi: 10.1371/journal.pone.0250919 (PMC8211200; doi:10.1371/journal.pone.0250919)
Supplement: Attachment — Submitted filename: PONE-D-19-35850_reviewer+F.pdf [file pone.0250919.s004.pdf]

PLOS ONE

Challenges to nutrition management among patients on antiretroviral therapy in primary health centers' in Addis Ababa, Ethiopia; A phenomenology study

--Manuscript Draft--

**Manuscript Number:**	PONE-D-19-35850
**Article Type:**	Research Article
**Full Title:**	Challenges to nutrition management among patients on antiretroviral therapy in primary health centers' in Addis Ababa, Ethiopia; A phenomenology study
**Short Title:**	Challenge to nutritional management
**Corresponding Author:**	Helen Ali, MSc<br>Wolaita Sodo University<br>Wolaita Sodo, ETHIOPIA
**Keywords:**	Nutritional Management<br>HIV<br>ART<br>Phenomenology
**Abstract:**	Introduction<br>Nutritional management is a fundamental practice to the concern of all patients infected with human immunodeficiency infection (HIV). The nature of HIV/AIDS and malnutrition impacts are interlocked, intensify one another in a limitless manner.<br>Objective: This study aimed to explore challenges in nutrition management among people living with HIV on antiretroviral therapy (ART) in primary health centers in Addis Ababa, Ethiopia.<br>Methods and Materials: - A hermeneutic (interpretive) phenomenological study design was used. The study used in-depth semi-structured interviews to describe lived experiences among adult patients aged 18 and above and participants were selected purposively until saturation of idea reached. Scientific rigor and trustworthiness like credibility, transferability, dependability, and conformability were secured in each respective step. Verbatim transcription followed by translation and re-reading of the data has been secured to immerse in the data. The data has been analyzed through inductive thematic analysis assisted by NVIVO version 12 pro software.<br>Result : - Nutrition management challenges for HIV patients have been described using six major themes. The major themes were acceptance of the disease and the health status, facilitators and barriers of treatment adherence, behavioral changes in eating patterns, and experience of food security issues, nutrition knowledge, and support. The themes have explained how patients on ART have been challenged to manage their nutrition ever since their diagnosis. Among these, food security issue among ART patients has been found to be the leading major theme for other themes in which other themes can be controlled with<br>Conclusion and recommendation: - Taken together this research found that there was a huge challenge beyond taking medications among patients on ART. There should be a need to increase food security issues and various approaches in a different hierarchy of implementation.
**Order of Authors:**	Helen Ali, MSc
	Kassa Daka
	Befekadu Bekele
	Mengistu Meskele
**Additional Information:**	
**Question**	**Response**
**Financial Disclosure**<br><br>Enter a financial disclosure statement that describes the sources of funding for the	Norwegian agency of development (NORAD), Wolaita Sodo University College of Health Science has funded this research as part of Master's Degree research.

work included in this submission. Review the submission guidelines for detailed requirements. View published research articles from *PLOS ONE* for specific examples.<br><br>This statement is required for submission and **will appear in the published article** if the submission is accepted. Please make sure it is accurate.<br><br>**Unfunded studies**<br>Enter: *The author(s) received no specific funding for this work.*<br><br>**Funded studies**<br>Enter a statement with the following details:<br>• Initials of the authors who received each award<br>• Grant numbers awarded to each author<br>• The full name of each funder<br>• URL of each funder website<br>• Did the sponsors or funders play any role in the study design, data collection and analysis, decision to publish, or preparation of the manuscript?<br>• **NO** - Include this sentence at the end of your statement: *The funders had no role in study design, data collection and analysis, decision to publish, or preparation of the manuscript.*<br>• **YES** - Specify the role(s) played.<br><br>* typeset	
**Competing Interests**<br><br>Use the instructions below to enter a competing interest statement for this submission. On behalf of all authors, disclose any competing interests that could be perceived to bias this work—acknowledging all financial support and any other relevant financial or non-financial competing interests.<br><br>This statement **will appear in the published article** if the submission is accepted. Please make sure it is accurate. View published research articles from *PLOS ONE* for specific examples.	The authors have no competing interests

**NO authors have competing interests**<br><br>Enter: *The authors have declared that no competing interests exist.*<br><br>**Authors with competing interests**<br><br>Enter competing interest details beginning with this statement:<br><br>*I have read the journal's policy and the authors of this manuscript have the following competing interests: [insert competing interests here]*<br><br>* typeset	
**Ethics Statement**<br><br>Enter an ethics statement for this submission. This statement is required if the study involved:<br><br>• Human participants<br>• Human specimens or tissue<br>• Vertebrate animals or cephalopods<br>• Vertebrate embryos or tissues<br>• Field research<br><br>Write "N/A" if the submission does not require an ethics statement.<br><br>General guidance is provided below. Consult the submission guidelines for detailed instructions. **Make sure that all information entered here is included in the Methods section of the manuscript.**	Ethical approval and clearance were obtained from the Institutional Review Board of Wolaita Sodo University under Ref No: WSU335/334/11(S1 File) (18 May 2019). Formal permission was obtained from Addis Ababa Health Office to the respective health centers where the study was conducted.<br>The participants were reassured that none of the real identities would be revealed. All questions and concerns were addressed before signing the informed consent. Participants were informed that there was no direct benefit from this study except an opportunity to talk freely about their condition, which could be either a distressing or helpful experience. Also, they were informed the expected duration of interview(Maximum of 50 minutes).<br>The location ensured the participant's privacy and was mutually agreed upon. The participants were asked if they had any questions after completing the interview question guides. Informed written consent was a sign. The interview participants have the right to withdraw from the study at any time. If they did withdraw, no penalty, as well as the service they were getting, was not affected. They may have refused to answer any question or have the recording device stopped. If they did, the information would still be used unless they withdrew altogether.

Powered by Editorial Manager® and ProduXion Manager® from Aries Systems Corporation

**Format for specific study types**<br><br>**Human Subject Research (involving human participants and/or tissue)**<br>• Give the name of the institutional review board or ethics committee that approved the study<br>• Include the approval number and/or a statement indicating approval of this research<br>• Indicate the form of consent obtained (written/oral) or the reason that consent was not obtained (e.g. the data were analyzed anonymously)<br><br>**Animal Research (involving vertebrate animals, embryos or tissues)**<br>• Provide the name of the Institutional Animal Care and Use Committee (IACUC) or other relevant ethics board that reviewed the study protocol, and indicate whether they approved this research or granted a formal waiver of ethical approval<br>• Include an approval number if one was obtained<br>• If the study involved *non-human primates*, add *additional details* about animal welfare and steps taken to ameliorate suffering<br>• If anesthesia, euthanasia, or any kind of animal sacrifice is part of the study, include briefly which substances and/or methods were applied<br><br>**Field Research**<br><br>Include the following details if this study involves the collection of plant, animal, or other materials from a natural setting:<br>• Field permit number<br>• Name of the institution or relevant body that granted permission	
**Data Availability**<br><br>Authors are required to make all data underlying the findings described fully available, without restriction, and from the time of publication. PLOS allows rare exceptions to address legal and ethical concerns. See the PLOS Data Policy and FAQ for detailed information.	Yes - all data are fully available without restriction

A Data Availability Statement describing where the data can be found is required at submission. Your answers to this question constitute the Data Availability Statement and **will be published in the article**, if accepted.<br><br>**Important:** Stating 'data available on request from the author' is not sufficient. If your data are only available upon request, select 'No' for the first question and explain your exceptional situation in the text box.<br><br>Do the authors confirm that all data underlying the findings described in their manuscript are fully available without restriction?	
**Describe where the data may be found in full sentences. If you are copying our sample text, replace any instances of XXX with the appropriate details.**<br><br>• If the data are **held or will be held in a public repository**, include URLs, accession numbers or DOIs. If this information will only be available after acceptance, indicate this by ticking the box below. For example: *All XXX files are available from the XXX database (accession number(s) XXX, XXX.*).<br>• If the data are all contained **within the manuscript and/or Supporting Information files**, enter the following: *All relevant data are within the manuscript and its Supporting Information files.*<br>• If neither of these applies but you are able to provide **details of access elsewhere**, with or without limitations, please do so. For example:<br><br>*Data cannot be shared publicly because of [XXX]. Data are available from the XXX Institutional Data Access / Ethics Committee (contact via XXX) for researchers who meet the criteria for access to confidential data.*<br><br>*The data underlying the results presented in the study are available from (include the name of the third party*	Hence this is a qualitative study, transcripts of the data are available and we are happy to provide when requested.

and contact information or URL).<br>• This text is appropriate if the data are owned by a third party and authors do not have permission to share the data.<br><br>* typeset	
Additional data availability information:	

Manuscript

Click here to access/download;Manuscript;PLOS_Manuscript_Helen_FINAL

Challenges to nutrition management among patients on antiretroviral therapy in primary health centers' in Addis Ababa, Ethiopia; A phenomenology study

Helen Ali [1*], Kassa Daka, [2], Befekadu Bekele[3], Mengistu Meskele[4]

[1,2,3,4] School of Public Health, Wolaita Sodo University, Wolaita Sodo, Ethiopia

Corresponding Author: [1]*E-mail Address: bravohelu@gmail.com P.O.Box: 138, Phone number: +251-909679708

Abstract

Introduction

Nutritional management is a fundamental practice to the concern of all patients infected with human immunodeficiency inf[illegible]on (HIV). The nature of HIV/AIDS and malnutrition impacts are interlocked, intensify one another in a limitless manner.

Objective: This study aimed to explore challenges in nutrition management among people living with HIV on antiretroviral therapy (ART) in primary health centers in Addis Ababa, Ethiopia.

Methods and Materials: - A hermeneutic (interpretive) phenomenological study design was used. The study used in-depth semi-structured interviews to describe lived experiences among adult patients aged 18 and above and participants were selected purposively until saturation of idea reached. Scientific rigor and trustworthiness like credibility, transferability, dependability, and conformability were secured in each respective step. Verbatim transcription followed by translation and re-reading of the data has been secured to immerse in the data. The data has been analyzed through inductive thematic analysis assisted by NVIVO version 12 pro software.

Result: - Nutrition management challenges for HIV patients have been described using six major themes. The major themes were acceptance of the disease and the health status, facilitators and barriers of treatment adherence, behavioral changes in eating patterns, and experience of food security issues, nutrition knowledge, and support. The themes have explained how patients on ART have been challenged to manage their nutrition ever since their diagnosis. Among these, food security issue among ART patients has been found to be the leading major theme for other themes in which other themes can be controlled with

Conclusion and recommendation: - Taken togeth[illegible]his research found that there was a huge challenge beyond taking medications among patients on ART. There should be a need to increase food security issues and various approaches in a different hierarchy of implementation.

Keyword: - Nutrition management, HIV, ART, phenomenology

Background

Nutritional management is a fundamental practice to the concern of all patients infected with human immunodeficiency infection (HIV) like the idea of the disease requires a multidisciplinary way to deal with it [1].

Energy requirements in HIV patients are going to increase by 10% to keep up body weight and physical movement in asymptomatic HIV-infected people. During symptomatic HIV, and in this manner during AIDS, energy prerequisites increment by around 20% to 30% to maintain body weight [2]

Nutrition Management covers issues of food insecurity and malnutrition which in turn poses challenges to antiretroviral treatment (ART) adherence in resource-poor settings and is often cited by PLWHA and care providers at health centers serving for accessing and adhering to ARV treatment [3].

People living with HIV/AIDS would experience different in managing their nutrition which in turn very helpful and appropriate for better construction of principles and moreover, promoting nutritional intervention and effective nutritional support is crucial to ensuring optimal ART uptake, adherence, and outcomes[6]

In the Global overview, over 800 million individuals are chronically suffering from malnutrition specifically undernutrition, and more than 35 million individuals are living with human immunodeficiency infection (HIV) and incidence of HIV have been accounted for everywhere throughout the world, most of the cases have been accounted for in low-and middle-income countries, especially in Sub-Saharan Africa (SSA). In Ethiopia, 1.5% of young individuals aged 15– 49 are infected with HIV[7,8].

Malnutrition is significantly interrelated with HIV which has brought bottleneck predominantly in Sub Saharan Africa and generally all over the world. HIV influences the wellbeing prestige of a person. Moreover, the socio-economic wellbeing of personalities infected with HIV and it also affects the economic growth of a country which in turn affects the development of a country [9,10].

Ethiopia is one of the countries that strike strongly by HIV infection within spitting reserve of the absence of concrete nutrition and it occurs in extraordinary structures and impacts various people either beforehand or inside the season of ART. Even though nutrition intervention thought to be given by trained counselors, a study conducted in Northwest Ethiopia revealed that dietary counseling was found to be not practiced [11]which most of the settings may share the same experience or not even providing the service.

The seriousness of malnutrition among PLWHA on ART is continually escalating and the interventions are provided by the health providers for better management, unexplored lived experience of patients on ART is found to be comprehended and right straight to the strength of nutrition maintenance of PLHWA. Besides, challenges in managing nutrition among patients should certainly be justified and extensively considered for identifying the strong gap and go through the right track when there are alarming conditions that are being the critical point in prolonging the life of PLWHA. Therefore, this study aimed to assess challenges to nutrition management among patients on ART in primary health centers' in Addis Ababa, Ethiopia.

Methods and materials

Study area and setting

This study conducted in Addis Ababa, a capital city of Ethiopia. According to the 2007 E.C Census Addis Ababa City has a total population of 2,738,248, where 48% are males and 52% are females. The total number of health centers in Addis Ababa is 119 and the average allocation of Health centers in eight health centers per sub-city according to the population coverage. In a total of nine regions, Addis Ababa is the second-highest city from the overall country with HIV prevalence (3.4%) followed by Gambella (CSA and ICF, 2018).

Research Design

Phenomenological study design has been conducted with consideration of Interpretative Phenomenological Analysis (IPA) which implies primarily works with transcripts of semi-structured interviews, which has been analyzed case by case and has been converted into a narrative account where the researcher's analytic interpretation is presented in detail and have supported with verbatim extracts from participants. The design attempted to use personal, in-depth detail derived from individual interviews among patients, and open-ended questionnaires among healthcare providers to describe their experiences of barriers or challenges to HIV-

related nutrition management.

Inclusion Criteria

Participants, who volunteered to participate in this study were at least 18 years old or above at the time of diagnosis (it hoped to control help the recall bias), speak and understand Amharic, permanent reside in Addis Ababa, well aware of their HIV status, and be willing to consent and speak about their experience, had at least prior exposure to severe weight loss secondary to malnutrition and recovered (graduated) with the help of supplemental foods provided at the health center. Participants lived at least one year with HIV with a maximum year of ten years. However, experiences could be different based on the number of years that the patients have. Meaning, a longer period a patient is with the disease the essence might be different.

Exclusion Criteria

Exclusion criteria included patients with psychiatric illness as it is destructive to the interview protocol

Data Collection Procedure

Semi-structured interview guides were provided for open-ended questions so that all aspects can be addressed. Questions were drafted based on the different phenomenon of participants by the researcher. Before the actual data collection, the data collector (the researcher) piloted the instruments with five from ART patients. Adjustments were made for flow, content, terms used, prompts, and instructions. Tools were translated into local languages as necessary which is in the Amharic language.

Thus it has helped to have a great detail of the phenomenon by following probes to follow a route of an investigation initiated by the participant. An interview meeting was scheduled at a time and place that is suitable for the participant as it was a separated room next to the examination and follow up the room. Interview was conducted by the primary auther. Participants were selected purposively until saturation of idea reached.

Audio reecordind and note taking were made. There were two audio-tape recorders in case of equipment failure. After all the questions were addressed, the researcher asked the participants if there was anything additional they wanted to discuss or mention. Participants in the study have also been welcomed if they thought of anything else after the interview. The body language, verbal, nonverbal cues were recorded using notes immediately after the interview. The researcher's reflection of the interview and labeled the audiotape with the record file name. The reflection allowed the researcher to bracket personal assumptions and reduce bias, however

in IPA study the researcher interpreting based on his own experience of the phenomenon. The interview was transcribed, which meant there would be a written version of the interview. Transcription was performed by the researcher and other transcribers. After the interview, transcription, and initial data analysis, the participant was contacted for transcription verification. The participant needed to verify that the transcription was exactly what she/he had said and verify the participant's description of her lived experience. A total of 28 in-depth interviews have been the most appropriate technique for data collection. The following tools have been used for the study.

Data Analysis

The recorded interviews were transcribed in Amharic, translated into English, and then managed using NVIVO version 12 pro software. The current study has summarized massive amounts of data with important features of the interview. As per Cress well description of six major steps in analyzing data in phenomenology, the current study has followed six major steps of analysis. First, the researcher managed the data by creating and organizing files so that they can be accessed easily for analysis. Second, the principal author reads and rereads the transcriptions, making notes in the margins identifying any sub-themes and wrote emerging ideas form baseline codes. Third, the researcher describes the personal experiences throughout the period, attempting to identify the core meaning or essence of the phenomena, the concepts, or themes were derived from the data. Fourth the data has been classified by developing relevant statements. The statements were grouped according to significance and meaning in the coding process. Step five was interpreting phase, which is a written description to answer “what happened?” and “how the phenomenon was experienced. Thus, the essence of the experience of the participants’ has been revealed. The final step was summarizing findings of the study, and this has been also supported with additional paragraphs which intend to interpret the meaning of the experience.

Scientific Rigor and Quality Assurance

Credibility

In the current study, this has been achieved by prolonged engagement, peer debriefing, member check, regarding prolonged engagement, the time the qualitative researchers spent in the field collecting data, observing, and interviewing to gain in-depth knowledge...

Dependability

This section is mainly the reliability of the study in which this study has shown in the data collection and data analysis process. The accurate documentation including all documents in

the final report, avoiding spelling and grammatical errors that will reduce the quality of work, and a detail description of producers is included.

Conformability

These results and interpretations were based supported by the collected data and also reflects the objectivity of the data.

Transferability

Transferability has been ensured by providing a detailed description of the study thereby providing opportunities for other readers to contextualize to another area.

Operational Definitions

Challenge of nutrition management: is a phenomenon of accessing and eating foods that help to keep maintain the optimum level of nutritional status of HIV patients on ART in which can help to perform regular activities.

Weight loss: is referred to in this study reduction of weight among patients with HIV on ART secondary to malnutrition when other weight loss factors were ruled out

PLHIV: - It refers to the people living with HIV on ART 18 and above years of age and diagnosed as a case of HIV as per the records and who are attending selected ART Centers for treatment in primary health centers

Ethical Considerations

Ethical approval and clearance were obtained from the Institutional Review Board of Wolaita Sodo University under Ref No: WSU335/334/11(**S1 File**) (18 May 2019). Formal permission was obtained from Addis Ababa Health Office to the respective health centers where the study was conducted.

The participants were reassured that none of the real identities would be revealed. All questions and concerns were addressed before signing the informed consent. Participants were informed that there was no direct benefit from this study except an opportunity to talk freely about their condition, which could be either a distressing or helpful experience. Also, they were informed the expected duration of interview(Maximum of 50 minutes).

The location ensured the participant's privacy and was mutually agreed upon. The participants were asked if they had any questions after completing the interview question guides. Informed written consent was a sign. The interview participants have the right to withdraw from the study at any time. If they did withdraw, no penalty, as well as the service they were getting,

was not affected. They may have refused to answer any question or have the recording device stopped. If they did, the information would still be used unless they withdrew altogether.

Result

Demographic Repre[illegible]tation

The study included 28 HIV patients on ART who lived in Addis Ababa. Twenty-eight ID participants were recruited from three governmental health centers. The participants' age varied from 29 to 61, with a mean age of 42 years. The participants reported that they were living with this disease from 2 years to 10 years. Although all the participants' were living in different Sub-city of Addis Ababa. Twenty-one of the women stated that they are Orthodox Christian by religion; 6 participants are Protestant Christian and only 1 Muslim. Their marital status was reflected with four status and 11 were married, 5 were divorced, 4 were single and 8 were widowed. Regarding their employment status, 8 of the 28 participants were unemployed, 2 were governmentally employed, 17 were employed in the private sector as Driver, Security, and waitress, and daily laborer and one was a commercial sex worker (Table 1**) (S2 File).**

Themes

Six major themes were identified from participants' testaments: Acceptance of disease and health status, facilitators and barriers of drug adherence, behavioral change in eating pattern, the experience of food security issues, nutrition knowledge and support were assembled as a major challenge in their life course ever since their diagnosis. Also, it shows the participant's extensional life worlds of space in controlling and maintaining their nutrition. These themes were not restricted to a specific participant because the experiences of a single participant could belong to more than one theme (Figure 1).

Theme I: Acceptance of disease and health status

This theme is concerned about how accepting a new phenomenon was a challenge to have better nutrition management. Participants voiced accepting the positive status was a challenge to have positive behavior towards nutrition. The phenomenon was beyond the support and education made by health professionals at the health center in this regard. As a result, patients were kept themselves from taking any medication and that lead them to health and physical inconveniences. The other factor which was mentioned by them was spirituality. For some, spirituality was mentioned as a restricting factor as they spent their time in the holy water without taking any medications and for others, their spirituality had helped them to accept their health status. Besides, participants stated their [illegible] seeking behavior was poor

which results in a denial of their positive results so that, they couldn’t manage their nutritional status.

> *"How could I start taking medication and eating foods if I can't accept my positive result ....for me…it was a terrible situation and I was born again when I start accepting the reality…I have started taking my foods and medications after that" Female Unemployed, Aged*

> *"...I went to holy water which is 30km far from Addis Ababa. When I was entered to the monastery I have just stopped all foods for the blessings. I took food…well it was "Kita" after one day I didn't take any medication at that time. I thought I could be cured and I suffered for 15 days…” Male self-employed*

As noted by the participants in the current study, an extended and challenging route to accepting one's diagnosis was common. Meaning, participants meant to highlights how accepting an HIV diagnosis can be a protracted and multifaceted progression for them. Moreover, their denial of the test result is prioritized and it hindered the acceptance of a new HIV diagnosis and participants' lack of a need for treatment until one is seriously ill. This study has found the difficulty of accepting and internalizing one's HIV status is frequently a challenge and is often a central reason for starting a proper eating pattern. Fear of stigma and other social discriminations could be the best explanation for their un acceptance behaviour at the time of their diagnosis. In addition, in the case of spirituality, participants meant to issue they used a means of sources to heal and cure of HIV/AIDS in which they believe holy water purifies whoever is touched by it and frees him from any disease.

Theme II: Facilitators and barriers of treatment adherence

This theme represents the anticipatory beliefs of how food, drug and patient's drug adherence is tempted to live healthy nutrition management. Participants stated facilitators and barriers for their nutrition management as their experience was interlinked one to the other to eat well. They stated whenever they are not taking the drugs they had difficulty eating their meals regularly. An important influence on their nutrition management was treatment know-how and passive tolerance of side effects of the treatment.

> *"Taking a medication appropriately is meant a lot for me…what I want to tell you about my drug is how it helped me to live healthily. I need to live healthily and work my regular activity. Nevertheless, I have started it lately, I know how I am benefited and surviving my health condition. Look…the drug is also a food that enables us to eat regular food…" Male Government employee*

Participants did not know how ART drugs and how they work actively, as a result, they are reluctant to adhere to the treatment. Moreover, participants' also stated that the drug had a direct and numerous effect on their daily eating pattern. Optimistic beliefs and tolerance for the side effects of a treatment given were explained as a moderator as it affects their food intake.

> *"I started eating and gaining weight after I have started the drug. I sleep well at night. I went out for work out and I earn at least a little and that is because I started to work" Male Driver "Previously when I started the medication it was 3 in kinds and that has to be taken every three hours …though it was very challenging but I have accepted it. I got no choice. I have to take the drug and when it is challenging for me what can I do? I just simply accept it" Male Unemployed*

The interviewees have asserted the drug as a food that helps to maintain their regular foods. Participants' commonly made a strong relationship between situations and felt about how it affects their nutrition management. They had compliant with their prescribed medications. Other participants, particularly those with other comorbid diseases, described taking medication inconsistently, have experienced a lot to manage their nutrition. For most of the participants, it seemed that almost all participants became tolerant to the side effects from time to time and stated as a facilitator and a barrier to achieving proper nutrition management.

Theme III: Behavioural change in eating pattern

Behavioral change in eating patterns is distinct from defining experiences, such that eating patterns in this theme captures the on-going and dynamic process affecting the quality of life of participants in the current study. It consists of factors influencing their nutrition management as they have changed their eating behavior. Of the factors, loss of cultural practice because of the changes they made and the use of supplemental foods as they failed to manage their nutrition. Participants expressed eating pattern changes and how that was a significant challenge for them to control their eating. Shared descriptions represented the behavioral change in eating pattern as an expedition, an on-going process that which they made blindly time to time, sometimes they people fail to recall what to do about it and learn as their situation were normal as a result they have done it blindly.

> *"…Of course, there was a change in my eating ever since my diagnosis. Yes…that was one of the stressing events so that one might not be able to think to manage his eating...I knew it… they changed their eating and I did. I ate food intentionally considering my status…HIV… but it was challenging because of my little understanding "Female unemployed*

> *"You have to take care of yourself but you are in the meantime going apart from your social food ceremonies...I ate something before three years in 'Meskel' festival but I was sick and taken to a health center the very next day" Female governmental employee*

Dietary changes in HIV patients is meant in the current study as a challenge to manage nutrition for HIV patients on ART. Meaning, participants were challenged to manage their nutrition because of the routine appetite problems. Besides, they have also reported the eating pattern change results in social isolation in festivals and ceremonies that people supposed to eat together. Participants also explained the challenge noticed in the commencing of supplemental foods at the health center. Meaning, it was given when patients found undernourished and should be supported by ready to use supplemental foods.

> *"Taking that .....what was that ....there is food which is packed the test is similar to a nut. It is very difficult to eat for some of us because It has a different test…but it was not easy to take though… It nauseates me yak…" Female Daily laborer Aged*

Theme IV: Experience of food security issues

Participates in this regard have raised two major aspects of their food security experience which are their purchasing power related to what is available in a nearby market and the need to travel to buy cheaper foods and groceries outside their compound. They have stated as they are suffered the issue to secure their food. It is also reported as a barrier to adhere to their drugs. Participants reported that the limited availability of food affects their lifestyle change. On the contrary, some participants have also discussed there is plenty of availability of food but their major problem is they cannot afford the food. They have stated the uncertainty of having sufficient food supplies in their nearby setting and market areas.

> *"It is difficult sometimes to talk about food when there is nothing you can do about it. How could I help to manage my nutrition when I got nothing to eat. You might not be able to find anything which you can afford to eat. Tell me how I can take my medication. But health professionals advised me so many times ...well, I wonder if he showed me where I can find that....it was very challenging."(Female Unemployed)*

> *"I sometimes feel how I am going to cope with things and how I can proceed living like this...I have been diagnosed with mental disturbances. I am worried about the foods around me and then how I am going to take my drug." (Female unemployed)*

> *"What can I say about my purchasing power? You might not have the chance to see what is important to you. You can't pay for what you want to eat rather you can only pay for what you can only eat." (Female self-employed)*

Participants meant to describe the challenges to nutrition management and how it related to drug adherence. It is stated and reported that financial limitations pose a barrier to follow a healthful diet and that is to the extension of unavailability of foods. The understanding of food insecurity is described as the' constrained or unstable availability or limited or unsure capacity to obtain appropriate food in a socially appropriate manner. Many People living with HIV absence access to sufficient amounts of nutritious foods in resource-limited contexts in which it presents auxiliary difficulties for anti-retroviral (ART) success. Some individuals are likely to be discontinued owing to an absence of appropriate nutrition which needed for use of ART leads to decreased food security.

Theme V: Knowledge of nutrition

Although a few participants seem to have better knowledge about nutrition and had a prior understanding of food and related issues, the majority of participants shared that they had poor knowledge and understanding of what is better for their eating behavior along the way. Participants explained the difficult situation to comprehend what they needed for meals. Their weight loss was attributed to their previous nutritional knowledge and understanding and they revealed that they had poor nutrition education experience at the health center. All participants described the need for nutrition education for HIV patients and shared that it felt like many people living with HIV have a misunderstanding about how they manage their nutrition.

> *"The physician told me that I have to take my drugs …but regarding what to eat I don't know…If he cannot advise me what to do with my eating, then it will be very difficult to me to understand because I am a daily laborer and I don't know the food details" Male Daily, laborer*

> *“I don't think they advise others too. My experience regarding this is very poor. I mean I never heard about what to eat from this health center. So, do you expect me to know and explain something about food and how to eat? (Laugh)…No" Female Waitress*

On the contrary, some participants struggle with accepting the need for food habits change when they are advised by health professionals. The struggle participants experienced were with supporting negative motivation as it crumpled their reputation for enduring change. Participants reported despite they believe health professionals to educate them about recommended dietary changes, they haven't received and even heard about the specific food types that have specific nutritional contents.

> *"No one will advise not to take any specific food items. There is nothing like that. They only ask your condition… " female unemployed*

Participants in this description meant to say how nutrition knowledge and poor education affect their nutrition management. Participants reported despite they believe health professionals to educate them about recommended dietary changes, they haven't received and even heard about the specific food types that have specific nutritional contents.

Theme VI: Support

Participants conveyed their lack of support from parents and the community. They identified that support in the home would facilitate their lifestyle change, including assistance with meal preparation. Meanwhile, they have also stated that they had adequate appliances in the home to support meal preparation. While some felt it would be a challenge to be isolated and a meal prepared separately for them, others described ways in which following special food preparation would be helpful for them. Participants have reported experience sharing between patients as it will help for patients who are newly diagnosed. They also reported this experience sharing has never happened throughout their experience.

> *"You need to have support from family. I can't deny this. There is nobody even at my home. I used to live with my little sister. She got married and left me alone. It was difficult. She has never seen me ever since her marriage. Because the social stigma is not decreased. I am a victim of that." Female self-employed*

> *" I believe it is important but you can't have such kind of chances. Maybe I remember a guy whom I met him in the waiting area and it was very difficult to start the conversation because they might not be interested in the discussion and nobody has helped me to have that culture." Male driver*

Regarding their interaction with health professionals, the majority of participants in the study were happy about the way health professionals received and treated them except nutritional and health education at the health centers. They have also stated their gender preference among health professionals. Meaning, the respondents described their support for the present study because it provides them hope for survival and overcoming the situation and stage of the disease. Some lately diagnosed individuals will discover it difficult to take the first step in HIV treatment. Adhering to the treatment and manage nutrition one should get support from the other side. The other side of support can be found from family, friends, social and also other HIV positive individuals who lived in the same condition. People will educate themselves by sharing experiences with other HIV positive patients. If they do not have support from those sides, it will not be possible for them to adhere to nutrition and the drug as well.

Discussion

This analysis identified six major themes associated with the challenges of nutrition management among ART patients. Major themes had a significant impact on ART patient's nutritional and changes in their routine life to meet the expectations. All of the identified factors were interrelated (Fig. 1). The identified themes were: acceptance of disease condition and health status (theme one), facilitators and barriers of treatment adherence (theme two), behavioral changes in the eating pattern (theme three), experiences of food security knowledge (theme five) and support (theme six). Moreover, the major theme which is experiences of food security issues is found to be interlinked one to the other bidirectional. It is, therefore, vital to controlling food insecurity to help achieve the other challenges of nutrition management. Besides, some of these themes are a controlling mechanism to overcome nutrition management.

Not all people find out well that they have an altered health status when they seek evaluation of an upsetting symptom. They may learn to know the illness conditions even though they have no symptoms and others might expect the positive results of their status and ready to accept the consequences. When a person decides to take a test on HIV, there are also different reasons for testing. Often people take tests even though they are asymptomatic because they fear their prior exposure to the disease. There is a way to increase the reason for testing beyond the health facility so that people will get tested and easily accept the test results. This has also been consistent and supported by other studies [12,13]. The current research finding suggests to pick out as a problem as it is one way to manage the experience forward. Even in this situation, it is helpful to explore such issues as the individual's motive to commence the medications indicated to treat the disease.

The present result on acceptance of disease condition and health status is consistent with another study finding that showed a majority of HIV positive individuals have a challenge on accepting their condition at the very beginning and sometimes will take a long to accept the situation. When patients have engaged in risky sexual behavior, such as inconsistent condom use, that promotes the spread of HIV[14]. Moreover, individuals are not aware of their echelons of sexual risk-taking behaviors. The majority of respondents perceived themselves as at no or low risk of HIV infection, while very few participants engaged in some form of risky sexual behavior such as unprotected sex and having multiple sexual partners.

Specifically, participants who perceived themselves at low risk were less likely to seek testing, regardless of risky reported sexual behavior [15].

The present study shows that people tested due to forceful reasons which they made them get tested and that lead them to experience challenges in accepting their Health Status. The forceful reasons that they commonly stated were known HIV illness and death of spouse, pregnancy & leaving the country. The current study is found to be consistent with other studies [16] which revealed the mentioned findings. The three major factors which have been stated as inhibiting for the utilization of Voluntary Counseling & Testing (VCT) service were fear of stigma and discrimination, fear of coping with positive HIV test result and high HIV risk perception. However, having a marriage plan and persuasion by health professionals was not found in this study as a cause for the participants' test. This is due to the reason that most participants were married and they didn’t mention why they get tested other than known HIV illness and death of their spouse. Participants voiced about the strong challenge in accepting the disease condition is high when they were pregnant as they are not ready for positive results [17]. This finding is consistent with other studies that revealed not having a plan to disclose the status of test results were negatively affect the acceptance of provider-initiated HIV testing in pregnant women [18].

The findings of this study regarding counseling suggest not that the pre and post counseling context changed the nature of counseling and the trend in both contexts is quite different. Participants in this study have voiced about the post counseling which needs a tremendous approach by the health professionals. Participants also were presented with some dilemmas to come and get counseled about how important the next step of their life is. These dilemmas were patterned strongly by educated participants as it leads them to poor linkage to the care and it is coherent with the findings of the study which was conducted by access to HIV counseling and testing in Ethiopia [19]. This research has shown that, as in the current study, there is inadequate post-test counseling along with other factors is listed as a factor for poor linkage. It is one of the main reasons for poor linkage. Moreover, notably in the current study, the issue of starting ART is more focused than post counseling and these findings may reflect the need for a systematic approach of counseling to address the issues depending on the different backgrounds of patients.

In the current study, religion is a factor for accepting the health and disease condition of a patient. The factor can be seen in two sections. Religion and spirituality is the basic element

for Ethiopians according to other studies [20] Patients stated that spirituality has helped them to cope with their problem because they will accept situations easily with the help of their spirituality and this finding has been seen consistent with the study conducted by [19] which showed as spirituality/religion helps people to cope with stressors, especially stigma/discrimination. Spiritual interventions utilizing the power of prayer and meditation and addressing spiritual struggles are underway. But on the contrary, the current study has also an idea of religion as a reason for not being able to start the medication early rather people with HIV prefer to go to the holy water and spend their time without starting any medication and eating well. Thus, they are prone to complicated situations and back to the health center with serious illness. Even though, there is a contradiction study that showed a willingness to begin ARV treatment was not significantly associated with the belief that prayer could cure HIV or with other religious factors. Refusal of ARV treatment was instead correlated with a lack of secondary schooling and a lack of knowledge about ARVs. This contradiction might be due to the setting of the study area and the social culture of participants [21].

The current study revealed that HIV patients who are on ART are suffering from different kinds of food security issues. The factors are interrelated with availability, affordability, and utilization of foods which they owe to eat. The food security issue is seemed to be major for all themes that patients felt comfortable when they are secured to manage the food availability, affordability, and utilization. Therefore, when designing to active life expectations and managing the drugs, it is important to consider awareness of individuals as they do not know how to survive and live with their living standards. They felt at risk of can't able to survive. These findings are supported by different studies that revealed malnutrition among ART patients [22-24]. Therefore, there should be nutrition care at the primary health center which may be a more appropriate location for all individuals to manage their nutritional status and what it necessitates.

Subthemes has been extracted with the support of the major themes. Themes are also being extracted related to challenges while taking medications and adherence. The main barriers of adherence discussed by the participants centered on treatment know-how, tolerance of side effects, drug-related routine appetite problems and drug availability. Social support (Theme 6) was found to relate to drug adherence. There is a need to adhere to the drug for a person to survive while living with HIV and thought to be perceived benefits of adherence. Subthemes

which has been discussed by respondents have been evaluated and need clear awareness creation across patients and health professionals regarding adherence to the treatment. The present study revealed participants have suffered a lot of adverse reactions of medications This also supported and coherent with a study conducted in Gonder that revealed that patient had a chance of facing three adverse effects from their ART regimen which is in contrast with a study done in Uganda, which reported an average of five side effects could be possible, either way, the two findings from a relative study setting revealed that ART drugs have side effects depending on its regimen [25]. In the current study, patients have reported repeatedly vomiting and gastrointestinal discomfort in contrast to these studies. This could probably be due to differences in patient’s reports of adverse effects and the settings of the study which is at the tertiary level in which patients come with the severe stage of the disease.

In this study, the critical idea which summarizes the theme was treatment knowledge treatment know-how, emotions and passive tolerance of side effects, nutritional advantages of the drug. Treatment knows how is low in the current study and this is inconsistent with a study conducted in Arsi [26]. These factors have been mentioned as a barrier for adhering to the treatment and result in a challenge of nutrition management. These findings might not be in line with other studies that stated distance as a cause for non-adherence. Those who accessed the health institution far away (>20 km) from their home were found to more likely use no adherence as a coping strategy than those who were nearby (≤20 km) this variation is might be due to the participants to the current study are using the nearby health centers because the setting is in Addis Ababa and also the study method [27].

Respondents had a change in their eating behavior ever since they have been told about their health status. The un-clarity about the composition of foods and what to eat leads them to have a variety of changes to their lives. These findings are consistent with many studies that they explore how the changes have been made According to participants their change influences their cultural eating practice due to the attention for their eating behavior change. Losing one's cultural practice might result from adopting a new culture. This has been seen in other studies when they assessed and stated as To change negative health behaviors, one must first identify and promote positive health behaviors within the cultural logic of its contexts [28]. HIV patients in this study are avoiding foods that they have been told to cause infections as they are immune-compromised. Foods that patients are taking is by avoiding the uncooked and cold foods as it causes infections and leads to the opportunistic disease [29].

On the other hand, supplemental foods are thought to be a new eating behavioral change in the current study as they are taking from health centers due to many of their undernutrition status and they have been benefited and survive using the supplemental. This finding is consistent with the need and use of supplemental foods for Patients on ART[30,31].

Nutrition knowledge is found to be the basis for all in the current study. As nutrition is basic for people with HIV and it is all about immunity and improving one's living condition. The more people are spending their time on nutrition the more they are a better fighter to their disease condition. According to the current study, participants do not have the basic concepts of nutrition and sought to have an approached education by health professionals at the primary clinic and nutrition understanding and knowledge gap has been seen low among HIV patients in many studies. This has been in line with other study findings such as a study conducted in Felege Hiwot referral Hospital [11] which revealed that dietary counseling were significant factors for nutritional Knowledge and sought to have nutrition education and counseling should be given by health care workers for patients on ART to improve their nutritional knowledge. However, in this study, it shows the significance of giving education to the level that they can practice. Educating how healthy a food does not change their practice rather educating to change their actual life is thought to be beneficial for patients who are living with HIV and a study conducted in Addis Ababa revealed that factors identified with a low adherence were a low level of education, poor knowledge is useful to adhere to food and nutrition programs [32].

Regarding support, experience sharing among ART patients, in this study, patients does not have the chance to discuss issues with other patients and they highly recommend the interventions to create their lives practical. There are studies that are consistent how the importance of conducting this kind of program which revealed the difficulty of disclosure is not easy and it is even very challenging for a couple to disclose their status and discuss situations(1). However, there are studies that contradict the experience sharing issues as it exacerbates social discrimination for people who are living with HIV [34,35].

Strength and limitation of the study

The study provided insight into the participant's lived experience with managing nutrition. Different factors influenced the nutrition management of patients which has been found by triangulating data with supporting methods. Thus, data and method triangulation can be the strength of this study as it is evidenced by using IDI with other supporting techniques. In that case, the current study credibility has been tried to secure and thus the study has several important strengths. The other major strength is qualitative studies are well suited to identifying challenges from the perspective of the patients. The use of an in-depth interview (IDI) approach permitted to discover a diversity of ideas and practices which most likely would not have been detected using a quantitative approach.

The limitation of the current study is quite shared with qualitative research which is particularly for IPA study. The researcher in this study has by default privilege to construe and report what interviewees predestined within the researcher's theoretical schemes. Using this method in the current study provides room for the concept that the people’s experiences integrate the researcher’s views. IPA also requires theme development by the researcher by repeated reading of the actual data. In addition, all the required steps have been secured throughout to confirm the trustworthiness of the findings.

Conclusion

Taken together this research found that there was a huge challenge of nutrition-management among ART patients beyond taking medications.Our study identified six themes. Acceptance of disease and the health status, facilitators and barriers of treatment adherence, eating behavioral change, experience of food security issues, nutrition knowledge,and support. Among these, food security issue among ART patients has been found to be the leading major theme for other themes.The controlling mechanism for nutrition management should be given the focus and aimed to a scheme line to address food insecurity. Also, there should be a practice-based teaching strategy in which HIV patients can demonstrate health eating habits.

Supporting information

S1 File. Ethical approval

S2 File. Table one

S3 File. English questionnaire

S4 File. Amharic Questionnaire

S5 File. COREQ check list

Acknowledgments

I would like to acknowledge Wolaita Sodo University, Ethiopia for the opportunity they created for this study. I would like to extend my gratitude for the Addis Ababa health office and the respective health centers for all aspects of unreserved support when I required. My acknowledgment also goes to data study participants and ART clinic coordinators in the respective health centers.

Authors' contribution

HA: Conceived the topic and drafted the study as part of her Master's degree study; **KD, BB , and MM**: supervised the protocol development and revisited the manuscript critically for important intellectual content. The four authors are involved in the analysis and design of the study and also they read and approved the final manuscript. **HA, KD, BB,** and **MM,** are agreed to be accountable for all aspects of the work.

Data sharing statement

Data for this study is available in transcripts and we are happy to share the data when requested.

Competing interests

All authors declare that they have no conflict of interest.

Funding

Norwegian agency of development (NORAD), Wolaita Sodo University College of Health Science has funded this research as part of Master's Degree research.

Author's information

HA:- has an MSc in Human Nutrition, Wolaita Sodo University in the Department of Human Nutrition and reproductive health, School of Public health, College of Health Science and Medicine, Wolaita Sodo University, Ethiopia.

KD: is associate professor, in the school of public health, college of health science and medicine, Wolaita Sodo University

BB: MPH in Health Education and is a lecturer in the school of public health, college of health science and medicine, Wolaita Sodo University

MM: is a Ph.D. candidate at the University of KwaZulu-Natal, South Africa; MPH in reproductive and family health, assistant professor, in the School of Public Health, College of Health Science and Medicine, Wolaita Sodo University, Ethiopia.

References

1. Siberry GK. Preventing and managing HIV infection in infants, children, and adolescents in the United States. Pediatrics in review. 2014;35(7):268.
2. Psomas CK, Philibert P, Kinloch S. Highlights from HIV Glasgow, 28–31 October 2018, Glasgow, UK. Journal of virus eradication. 2019;5(1):67.
3. Franke MF, Murray MB, Muñoz M, Hernández-Díaz S, Sebastián JL, Atwood S, et al. Food insufficiency is a risk factor for suboptimal antiretroviral therapy adherence among HIV-infected adults in urban Peru. AIDS and Behavior. 2011;15(7):1483-9.
4. Palar K, Derose KP, Linnemayr S, Smith A, Farías H, Wagner G, et al. Impact of food support on food security and body weight among HIV antiretroviral therapy recipients in Honduras: a pilot intervention trial. AIDS care. 2015;27(4):409-15.
5. Swaminathan S, Padmapriyadarsini C, Yoojin L, Sukumar B, Iliayas S, Karthipriya J, et al. Nutritional supplementation in HIV-infected individuals in South India: a prospective interventional study. Clinical infectious diseases. 2010;51(1):51-7.
6. Scarcella P, Buonomo E, Zimba I, Doro Altan A, Germano P, Palombi L, et al. The impact of integrating food supplementation, nutritional education and HAART (Highly Active Antiretroviral Therapy) on the nutritional status of patients living with HIV/AIDS in Mozambique: result from the DREAM programme. Igiene e sanita pubblica. 2011;67(1):41-53.
7. Gedle D, Gelaw B, Muluye D, Mesele M. Prevalence of malnutrition and its associated factors among adult people living with HIV/AIDS receiving anti-retroviral therapy at Butajira Hospital, southern Ethiopia. BMC Nutrition. 2015;1(1):5.
8. Idele P, Gillespie A, Porth T, Suzuki C, Mahy M, Kasedde S, et al. Epidemiology of HIV and AIDS among adolescents: current status, inequities, and data gaps. JAIDS Journal of Acquired Immune Deficiency Syndromes. 2014;66:S144-S53.
9. Cypher J. The process of economic development: Routledge; 2014.
10. Gezahegn M, Upadhyay M. The effect of HIV/AIDS on economic growth of Southern African countries. Asian Economic and Financial Review. 2014;4(9):1146.

11. Mengie GM, Worku T, Nana A. Nutritional knowledge, dietary practice and associated factors among adults on antiretroviral therapy in Felege Hiwot referral hospital, Northwest Ethiopia. BMC Nutrition. 2018;4(1):46.
12. Van Dyk AC. Client-initiated, provider-initiated, or self-testing for HIV: what do South Africans prefer? Journal of the Association of Nurses in AIDS Care. 2013;24(6):e45-e56.
13. Mutale W, Michelo C, Jürgensen M, Fylkesnes K. Home-based voluntary HIV counselling and testing found highly acceptable and to reduce inequalities. BMC public health. 2010;10(1):347.
14. Njeru MK, Blystad A, Shayo EH, Nyamongo IK, Fylkesnes K. Practicing provider-initiated HIV testing in high prevalence settings: consent concerns and missed preventive opportunities. BMC health services research. 2011;11(1):87.
15. Horter S, Thabede Z, Dlamini V, Bernays S, Stringer B, Mazibuko S, et al. "Life is so easy on ART, once you accept it": Acceptance, denial and linkage to HIV care in Shiselweni, Swaziland. Social Science & Medicine. 2017;176:52-9.
16. Hirut BM. Factors Influencing HIV Voluntary Counseling and Testing (VCT) Service Utilization among Youth of Hawassa Town: A Health Belief Model Approach, Southern Ethiopia. Journal of Science & Development. 2014;2(1):49-58.
17. Assefa Y, Damme WV, Mariam DH, Kloos H. Toward universal access to HIV counseling and testing and antiretroviral treatment in Ethiopia: looking beyond HIV testing and ART initiation. AIDS patient care and STDs. 2010;24(8):521-5.
18. Ismail H, Ali A. Pregnant women's satisfaction and comprehension level of information given during HIV Counseling and Testing for PMTCT in public health facilities in Addis Ababa. Ethiopian Journal of Health Development. 2011;25(2):126-34.
19. Bibiana N, Emmanuel P, Amos D, Ramsey Y, Idris A. Knowledge, attitude and factors affecting voluntary HIV counseling and testing services among women of reproductive age group in an Abuja Suburb community, Nigeria. Medical Journal of Zambia. 2018;45(1):13-22.
20. Olsen M, Jensen NK, Tesfaye M, Holm L. Conceptual equivalence of WHOQOL-HIV among people living with HIV in Ethiopia. Quality of Life Research. 2013;22(2):361-7.

21. Berhanu Z. Holy water as an intervention for HIV/AIDS in Ethiopia. Journal of HIV/AIDS & Social Services. 2010;9(3):240-60.

22. Berhe N, Tegabu D, Alemayehu M. Effect of nutritional factors on adherence to antiretroviral therapy among HIV-infected adults: a case control study in Northern Ethiopia. BMC infectious diseases. 2013;13(1):233.

23. Gebremichael DY, Hadush KT, Kebede EM, Zegeye RT. Food Insecurity, Nutritional Status, and Factors Associated with Malnutrition among People Living with HIV/AIDS Attending Antiretroviral Therapy at Public Health Facilities in West Shewa Zone, Central Ethiopia. BioMed research international. 2018;2018.

24. Tiyou A, Belachew T, Alemseged F, Biadgilign S. Food insecurity and associated factors among HIV-infected individuals receiving highly active antiretroviral therapy in Jimma zone Southwest Ethiopia. Nutrition journal. 2012;11(1):51.

25. Kyajja R, Muliira JK, Ayebare E. Personal coping strategies for managing the side effects of antiretroviral therapy among patients at an HIV/AIDS clinic in Uganda. African Journal of AIDS Research. 2010;9(3):205-11.

26. Dibaba B, Hussein M. Factors associated with non-adherence to antiretroviral therapy among adults living with HIV/AIDS in Arsi zone, Oromia. Journal of AIDS and Clinical Research. 2017;8(1).

27. Gelaw YK, Adugna B, Tsegaye AT, Melaku T, Kefale B. Coping Strategies for Adverse Effects of Antiretroviral Therapy among Adult HIV Patients Attending University of Gondar Referral Hospital, Gondar, Northwest Ethiopia: A Cross-Sectional Study. AIDS research and treatment. 2018;2018.

28. Airhihenbuwa CO, Ford CL, Iwelunmor JI. Why culture matters in health interventions: lessons from HIV/AIDS stigma and NCDs. Health Education & Behavior. 2014;41(1):78-84.

29. Hussen S, Belachew T, Hussein N. Nutritional Status of HIV Clients Receiving HAART: Its Implication on Occurrence of Opportunistic Infection. The Open Public Health Journal. 2017;10(1).

30. Olsen MF, Abdissa A, Kæstel P, Tesfaye M, Yilma D, Girma T, et al. Effects of nutritional supplementation for HIV patients starting antiretroviral treatment: randomised controlled trial in Ethiopia. Bmj. 2014;348:g3187.

31. Olsen MF, Tesfaye M, Kaestel P, Friis H, Holm L. Use, perceptions, and acceptability of a ready-to-use supplementary food among adult HIV patients

initiating antiretroviral treatment: a qualitative study in Ethiopia. Patient preference and adherence. 2013;7:481.

32. Kebede MA, Haidar J. Factors influencing adherence to the food by prescription program among adult HIV positive patients in Addis Ababa, Ethiopia: a facility-based, cross-sectional study. Infectious diseases of poverty. 2014;3(1):20.

33. Genet M, Sebsibie G, Gultie T. Disclosure of HIV seropositive status to sexual partners and its associated factors among patients attending antiretroviral treatment clinic follow up at Mekelle Hospital, Ethiopia: a cross sectional study. BMC research notes. 2015;8(1):109.

34. Feyissa GT, Abebe L, Girma E, Woldie M. Stigma and discrimination against people living with HIV by healthcare providers, Southwest Ethiopia. BMC Public Health. 2012;12(1):522.

35. Wolfe WR, Weiser S, Bangsberg D, Thior I, Makhema J, Dickinson D, et al. Effects of HIV-related stigma among an early sample of patients receiving antiretroviral therapy in Botswana. AIDS care. 2006;18(8):931-3.

 Click here to access/download;Figure;Fig 1.tiff

Figure 1: Thematic map of identified themes

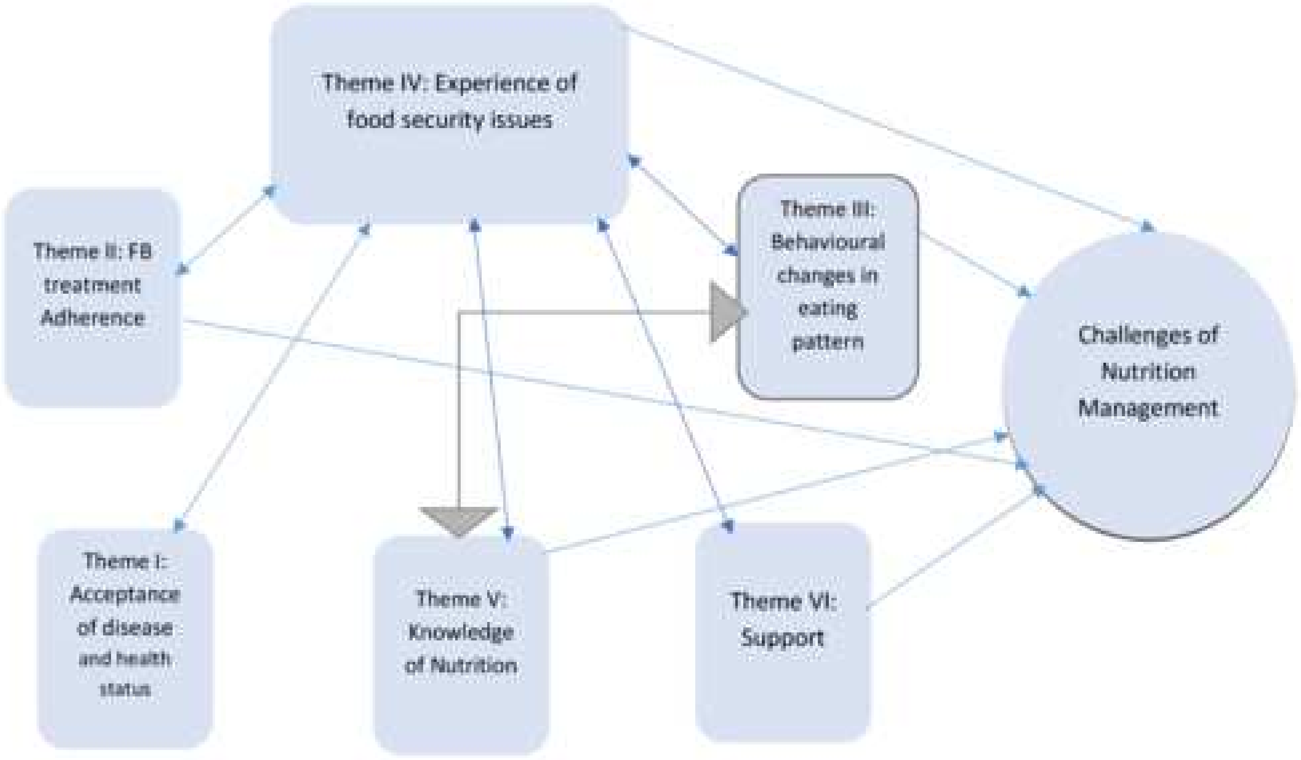

Table

Click here to access/download
Supporting Information
S2_Table.pdf

Questionnaire

Click here to access/download
Supporting Information
S3_Quesionnarie Amharic version.pdf

Questionnaire

Click here to access/download
Supporting Information
S4_Questionnaire English Version.pdf

COREQ check list

Click here to access/download
Supporting Information
S5- COREQ_Checklistf.pdf
