# Supplementary material for: Challenges to nutrition management among patients using antiretroviral therapy in primary health ‘centres’ in Addis Ababa, Ethiopia: A phenomenological study
Source: PLoS One. 2021 Jun 17;16(6):e0250919. doi: 10.1371/journal.pone.0250919 (PMC8211200; doi:10.1371/journal.pone.0250919)
Supplement: Attachment — Submitted filename: Response to Editors_7_25_2020.docx [file pone.0250919.s005.docx]

We acknowledge the editors for the valuable comments. We have corrected the manuscript as per the comment given.

1. Your ethics statement must appear in the Methods section of your manuscript. If your ethics statement is written in any section besides the Methods, please move it to the Methods section and delete it from any other section. Please also ensure that your ethics statement is included in your manuscript, as the ethics section of your online submission will not be published alongside your manuscript.

**Thank you very much. We have collected all the ethical issue in the method section only**

2. Please include a legend for figure 1.

**Thank you very much. We have included a legend for figure 1**

3. Please include a copy of Table 1 which you refer to in your text on page 8.

**Thank you very much. We have included Table 1 in the participant characteristics section of the manuscript**

4. Please upload a copy of Supporting Information File 1 which you refer to in your text on page 22.

**Thank you very much. We have uploaded supporting information File 1**

5. We note your Data Availability Statement currently reads as follows:

“Hence this is a qualitative study, transcripts of the data are available and we are happy to provide them when requested.”

We also note your following comments in your response to reviewers:

“We have included study's minimal data set as the underlying data used. This research was done on sensitive issues (ART clients) and it is not ethically permitted for us to deposit the data to the public. However, the transcripts we used and analyzed during the current study are in the main manuscript and all other necessary data sets are available from the corresponding authors on reasonable request."

Before we proceed with the review process, we’ll require some additional clarification to ensure your submission adheres to the PLOS ONE Data Availability policy (<https://journals.plos.org/plosone/s/data-availability>).

1.) Please confirm whether the minimal data set necessary to replicate your study’s findings is located in your manuscript and/or Supporting Information files. The minimal data set is defined as the data set used to reach the conclusions drawn in the manuscript with related metadata and methods, and any additional data required to replicate the reported study findings in their entirety (<https://journals.plos.org/plosone/s/data-availability#loc-minimal-data-set-definition>). This may include: a.) The values behind the means, standard deviations and other measures reported; b.) The values used to build graphs; c.) The points extracted from images for analysis.

2.) If the minimal data set is located in your manuscript and Supporting Information files, please confirm whether we may update your Data Availability Statement as follows:

“All relevant data are within the manuscript and its Supporting Information files.”

**We acknowledge the editor for the comment. We have edited the Data availability statement as**

**“All relevant data are within the manuscript and its Supporting Information files.”**

3.) If additional data is required to fulfill the minimal data set, and legal or ethical restrictions apply, please provide the following:
a.) A description of the data set(s),
b.) The reason for the restriction (i.e. sensitive patient information),
c.) The full name of the restricting institutional body (i.e. ethics committee, IRB),
d.) Detailed instructions for how other researchers can access the data, and
e.) Non-author contact information (preferably email) for a data access committee, ethics committee, or other institutional body to which data requests may be sent. Note that it is not acceptable for an author to be the sole named individual responsible for ensuring data access.

4.) Alternatively, you may upload your minimal anonymized dataset to a stable public repository. Please also provide the relevant URLs, DOIs, or accession numbers. For a list of acceptable repositories, please see <http://journals.plos.org/plosone/s/data-availability#loc-recommended-repositories>.

Your manuscript has been returned to your account. Please log on to PLOS Editorial Manager at <https://www.editorialmanager.com/pone/> to access your manuscript.
