# Supplementary material for: Challenges to nutrition management among patients using antiretroviral therapy in primary health ‘centres’ in Addis Ababa, Ethiopia: A phenomenological study
Source: PLoS One. 2021 Jun 17;16(6):e0250919. doi: 10.1371/journal.pone.0250919 (PMC8211200; doi:10.1371/journal.pone.0250919)
Supplement: Attachment — Submitted filename: Response for editor and reviewers.docx [file pone.0250919.s006.docx]

**Response for editor and reviewers**

Manuscript title: Challenges to nutrition management among patients using antiretroviral therapy in primary health ‘centres’ in Addis Ababa, Ethiopia: A phenomenological study

ID: PONE-D-19-35850R1

Date: 18 January 2021

Q. Language edition request by the editor

We thank the editor for this vital query. The language of our manuscript is now edited by native English language speaker, professor Roger Watson, who is helping researchers in the author aid service platform. He is now extensively revised the English twice, and we have corrected it. The modified files (edited twice) are indicated as a manuscript with track change 1 and 2.
