# Supplementary material for: Challenges to nutrition management among patients using antiretroviral therapy in primary health ‘centres’ in Addis Ababa, Ethiopia: A phenomenological study
Source: PLoS One. 2021 Jun 17;16(6):e0250919. doi: 10.1371/journal.pone.0250919 (PMC8211200; doi:10.1371/journal.pone.0250919)
Supplement: Attachment — Submitted filename: Response for editors_ 15 March 2021.docx [file pone.0250919.s007.docx]

**Response for editor and reviewers**

Manuscript title: Challenges to nutrition management among patients using antiretroviral therapy in primary health ‘centres’ in Addis Ababa, Ethiopia: A phenomenological study

ID: PONE-D-19-35850R1

Date: 18 January 2021

Q. Language edition request by the editor

We really thank the editor for this vital query. The language of our manuscript is now edited by native English language speaker who is helping researchers in the author aid service platform. The modified files are indicated as a manuscript with track change.
